# Involvement of the Anterior Commissure in Early Glottic Cancer (Tis-T2): A Review of the Literature

**DOI:** 10.3390/cancers11091234

**Published:** 2019-08-23

**Authors:** Martine Hendriksma, Elisabeth V. Sjögren

**Affiliations:** Department of Otorhinolaryngology, Head & Neck Surgery, Leiden University Medical Center, 2300 RC Leiden, The Netherlands

**Keywords:** anterior commissure, early glottic cancer, radiotherapy, laser surgery, oncological outcomes, survival

## Abstract

*Background:* The impact of the anterior commissure (AC) involvement on prognosis in laryngeal cancer remains a topic of discussion with inconsistent results in the literature. This review examines AC involvement as a prognostic factor in patients with early glottic cancer (Tis–T2) treated with radiotherapy or transoral laser microsurgery (TLM). *Methods:* A systematic literature search was performed. Due to the heterogeneity of the data, no meta-analysis was implemented. Weighted averages were calculated if the appropriate data were extractable. *Results:* Thirty-four studies on radiotherapy and 23 on TLM fit the inclusion criteria. The majority of studies for both radiotherapy (67.7%) and TLM (75.0%) did not report a significant impact on oncological outcomes. Weighted averages were slightly lower in patients with AC involvement. The two studies that applied a more detailed classification showed a significant impact on the amount of AC involvement. *Conclusions:* Binary variables (yes/no) for AC involvement lead to inconsistent results. Studies that use more detailed classifications of the AC show that there is a significant impact on the outcome. To further elucidate the role of the AC, detailed stratification of tumors involving the AC need to be investigated in further studies for both treatment modalities.

## 1. Introduction

Although it is widely acknowledged that the involvement of the anterior commissure (AC) in early glottic cancer (Tis–T2) can have negative impacts on outcomes, the extent of the impact remains a topic of discussion with inconsistent results reported in the literature. Some studies show a significant association between the AC and a higher recurrence rate, whereas others do not.

**Figure 1:**
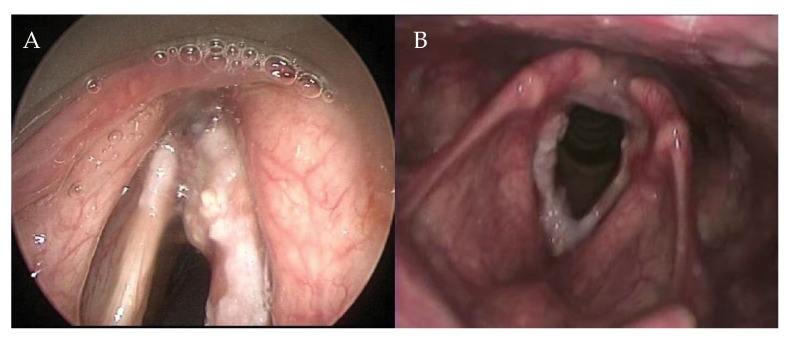
Extension in the anterior commissure. (**A**) Fiber endoscopic view during outpatient examination; (**B**) Endoscopic examination of the same patient in anesthesia.

The AC is a complex anatomical subsite of the larynx, which encompasses different structures such as Broyles ligament, membranes, muscles, perichondrium, and the thyroid cartilage, and has a close relationship with the visceral structures surrounding it. Therefore, the AC has to be considered as a 3D structure and not as a point location (Figure 1). Rucci et al. defined the AC—on the basis of embryonic development—as the area of the glottis situated anteriorly between the vocal folds that extends in a vertical direction, both upwards and downwards [1]. It is rarely the site of origin of glottic cancer [1], but it is often involved in anterior lesions spreading from left to right, and from inferior to superior. Furthermore, due to its close proximity to the visceral spaces of the larynx (pre-epiglottic space, paraglottic space, and cricothyroid membrane), it has been argued that microscopic spread to these spaces may affect local control [2,3,4].

The purpose of this study was to perform a systematic review of studies that have investigated the involvement of the AC as a prognostic factor, with the aim of answering the following question: Is the involvement of the AC a prognostic factor in patients with early glottic cancer (Tis–T2) treated with radiotherapy or transoral CO_2_ laser microsurgery (TLM)?

## 2. Methods

### 2.1. Search

A systematic search was performed on 7 January 2019 on PubMed. The search strategy was conducted with a combination of the following keywords: laryngeal cancer, radiotherapy, and transoral laser microsurgery. For these keywords, all synonyms were used.

### 2.2. Inclusion Criteria and Data Extraction

For studies to be included, they had to be on adult patients with glottic squamous cell carcinoma staged as Tis, T1, or T2, treated with radiotherapy or TLM, to investigate the involvement of the AC as a prognostic factor, and be published between 1998 and 2018 in English. Also, a clear distinction had to be made, within the studies, between tumors that did and those that did not involve the AC to test this variable. Studies concerned with recurrent cases and studies reporting on less than 10 patients were excluded. Full-text versions of the included studies were reviewed for oncological outcomes. The primary endpoint was 5-year local control (LC) of tumors, with or without the involvement of the AC, calculated by the Kaplan-Meier or Cox regression method. Other oncological outcomes of interest were overall survival (OS), disease-specific survival (DSS), and laryngeal preservation (LP). During the extraction of data, papers that did not report LC were excluded. After the full-text screening, all papers were checked for relevant citations.

### 2.3. Statistical Analyses

Due to the heterogeneity of the data, no meta-analysis was performed. If data were extractable, weighted averages of the data were calculated for the separate tumor groups: T1, T2, and T1–T2.

## 3. Results

### 3.1. Search

**Figure 2:**
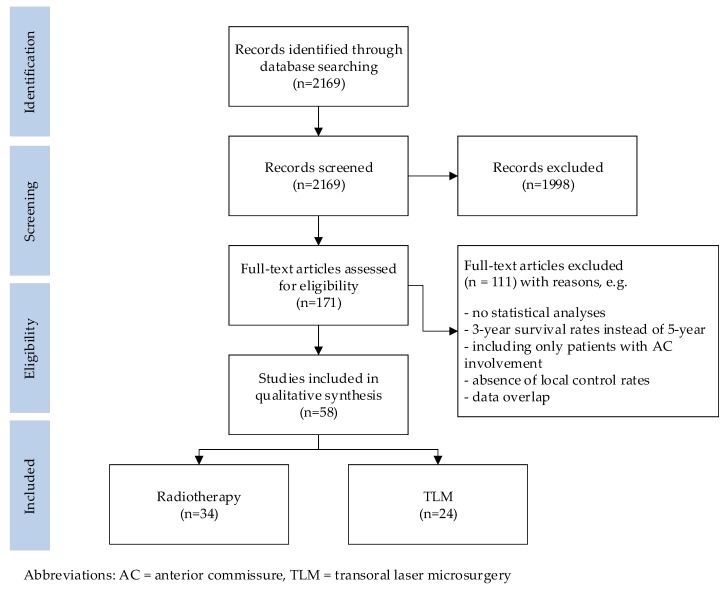
Flow diagram illustrating the searching and selection procedure.

The results of the search are summarized in Figure 2. The initial literature search yielded 2169 citations, of which the title and abstract were screened. This identified 171 publications that underwent a full-text review. Of these, 34 publications on radiotherapy and 24 on TLM met the inclusion criteria. Reference cross-checking did not identify additional papers.

### 3.2. Study Characteristics

All 58 studies included in this review were published in peer-reviewed journals. Only one prospective randomized study, which was on radiation therapy, was identified [5]. All other publications had a level of evidence classified as B [6]. Most of the studies reported outcomes for early glottic cancer, grouping Tis–T2 tumors together, with only a few studies focusing on T1 or T2 tumors separately. The 34 radiotherapy studies included 9656 patients, of which 3930 patients (40.7%) had involvement of the AC. The 24 TLM studies included 3958 patients, of which 1169 patients (29.5%) had involvement of the AC.

In the radiotherapy studies, different treatment protocols were applied, and different techniques were used (conventional, accelerated, hyperfractionated, hypofractionated, and intensity-modulated radiation therapy), with doses varying between 60 and 78 Gy. Administration schedules varied from once daily, five times per week to twice daily, six times per week. Some studies applied elective neck irradiation, and some administrated a bolus in patients with AC involvement. In 14 studies (41.2%) LC rates were not presented for the AC separately [7,8,9,10,11,12,13,14,15,16,17,18,19,20]. These studies only presented *p*-values, hazard ratios (HR), or odds ratios.

Most studies on TLM classified resections according to the European Laryngological Society (ELS) classification system [21,22]. Two studies [23,24] performed resections according to the principles proposed by Steiner and Ambrosch [25], and in three studies, the resections were not further specified [26,27,28]. In six studies (25.0%), LC rates were not presented for the AC separately [26,29,30,31,32,33].

In both the radiotherapy and TLM studies, the follow-up time varied. In radiotherapy studies, the follow-up time ranged between a median of 33 and 147 months, and in TLM studies follow-up time ranged between a mean of 24.2 and 84 months. Characteristics of the included studies are presented in Table 1 for radiotherapy and in Table 2 for TLM.

### 3.3. Local Control

In 23 out of 34 (67.6%) studies in the radiotherapy group, AC involvement did not have a significant impact on LC [5,7,8,9,11,13,14,15,16,17,18,20,34,39,40,41,42,44,46,48,49,51], and in 10 studies (29.4%), it did have a significant impact [10,19,35,36,37,43,45,47,50,52]. One study (2.9%) concluded that the AC was a predictive factor for LC in T1 tumors, but not in T2 tumors [38]. In the TLM studies, 18 out of 24 (75.0%) studies did not identify the AC involvement as a significant factor for LC [3,23,27,29,30,32,33,53,55,56,57,58,60,61,62,63,64,65], and two studies (8.3%) did [24,26]. One study (4.3%) concluded that the involvement of the AC was a predictive factor for LC in T1a tumors, although this was not the case in T1b or T2 tumors [28]. Three studies (12.5%) presented a more detailed classification of the AC involvement and concluded that the AC involvement had a significant impact on the AC [31,54,59]. One of these studies showed that in its binary approach (yes/no), the AC involvement did not have a significant impact on the AC, whereas it did in its more detailed classification [54]. 

Table 3 summarizes the weighted averages for the different tumor stages (T1, T2, T1–T2). Nineteen radiotherapy (55.8%) studies and 9 TLM (37.5%) studies could be included in the weighted averages. In T2 tumors treated with TLM, patients with involvement of the AC had a slightly higher 5-year LC rate than patients without involvement of the AC. In all the other groups, tumors with involvement of the AC resulted in a lower 5-year LC rate. 

### 3.4. Overall Survival, Disease Specific Survival, and Larynx Preservation

Four of the radiotherapy studies (11.8%) presented the 5-year OS [8,20,42,43]. The involvement of the AC did not have a statistically significant impact on any of these studies. Seven studies (20.6%) presented the 5-year DSS [8,9,10,16,20,35,38]. In two of these, the involvement of the AC had a statistically significant impact [10,38]. None of the radiotherapy studies presented the 5-year LP rates. 

Ten of the TLM studies (41.7%) presented the 5-year OS [23,24,26,28,32,54,58,59,61,65]. The involvement of the AC had a statistically significant impact for one of these for patients with T1b and T2 tumors, but not for T1a tumors [23]. In seven studies, no significant impact was found [24,26,28,32,58,59,61], and in two studies, the impact on OS was not reported [23,54]. Six studies (25.0%) presented the 5-year DSS. Two of these studies showed a statistically significant impact of AC involvement on DSS [53,58], and three studies did not [3,26,59]. In one study, the impact of the AC was not reported [54]. Ten studies (41.7%) presented the 5-year LP rate. Six studies did not show a statistically significant impact of AC involvement on laryngeal preservation [3,26,53,56,59,64], whereas one study did [58]. In two studies, the impact of AC involvement on LP was not reported [23,28]. One study presented a binary approach (yes/no) as well as a more detailed classification. The first showed no significant impact on the AC, whereas the latest identified a significant impact on the involvement of the AC related to the amount of involvement of the AC [54].

## 4. Discussion

Both radiotherapy and TLM are well–established treatment modalities for early glottic cancer involving the AC. Although it is widely acknowledged that involvement of the AC can have a negative impact on outcome, results reported in the literature on the impact of AC involvement have been inconsistent. In this review, we found that most studies—both for radiotherapy and TLM—do not report a significant impact of AC involvement on LC, OS, DSS, and LP. 

Although the results and the manner of reporting in the included studies were too heterogeneous to perform a formal meta-analysis, we did calculate weighted averages for T1 and T2 tumors separately and for T1–T2 tumors together from the papers that provided 5-year LC rates for the tumors with or without the involvement of the AC. On this basis, only 19 radiotherapy (55.9%) studies and 9 TLM (37.5%) studies could be included in the weighted averages. These weighted averages showed that the involvement of the AC leads to a slightly higher recurrence rate after treatment with both RT and TLM. However, as stated, this is no formal meta-analysis, and, therefore, no definite conclusions can be drawn from these calculations. The varying results in the literature can be explained by variations in the clinical definition of the AC area, and in the detail of the clinical, endoscopic, and radiologic evaluation of the lesion in the preoperative setting, the distinctive features and limitations of each therapeutic modality, the biological behavior of the tumor, and variations in the rigor of the follow-up policy. Due to these factors, combined with the complicated anatomy of the AC, the involvement of this subsite may very well be too complex to be included as a simple binary variable (yes/no) as it is in most publications. To try to draw some conclusions from the existing literature, it is, therefore, necessary to take a closer look at the data of individual publications and at the definition of involvement of the AC. In 1996, Rucci et al. proposed a new staging system of the anterior commissure, as there was no consideration of the AC involvement in the T stage of the TNM classification (Union for International Cancer Control-American Joint Committee on Cancer [66,67]). Rucci et al. classified the AC into four subgroups: AC0: patients without any involvement of the AC region; AC1: patients with involvement of the AC region on only one side of the midline, AC2: patients with involvement of the AC region that crosses the midline on only one part of the longitudinal extension of this region; AC3: patients with involvement of the whole AC region, on both sides of the midline [67]. They found that LC was significantly lower with the increase of the AC classification. They concluded that this AC classification was more reflective of prognosis than the TNM classification [67]. Since then, to our knowledge, every study utilizing this, or a similar classification of AC involvement into subgroups, has found a prognostic impact of increasing levels of AC involvement, with wider involvement leading to lower rates of LC or LP. Carta et al. did not show a statistically significant difference in LC rates between involvement and no involvement of the AC in patients with Tis–T2 tumors; however, they did find a statistically significant lower 5-year recurrence-free survival in the AC3 group when using Rucci et al.’s classification system. The AC3 group also showed a statistically lower 5-year LP rate [54]. Hoffmann et al. also found a significant difference in the 5-year disease-free survival in the AC3 group. However, they did not find a significant difference between the AC groups in terms of LP or DSS [59].

Recently, another classification was proposed by Piazza et al. [68]. They stratified six isoprognostic zones in early-intermediate tumors (T1–T3) treated with TLM according to the location and the extent of the tumor, describing different growth patterns and possible pathways of recurrence, and defined the role and limits of TLM as a single treatment modality. They concluded that the vertical extension across the AC leads to a decreased LC rate and lower LP rates in patients treated with TLM and that this location—with or without the involvement of the pre-epiglottic space (PES)—should be considered as a risk factor for TLM [68].

The classification of Piazza et al. regarding the AC is in line with earlier publications differentiating between the horizontal and vertical extension of the tumor [68]. In a recent review, Peretti et al. highlighted the importance of differentiating between tumors of the vocal cord affecting the AC in the horizontal plane against the vertical plane [69]. They defined several requirements when treating tumors involving the AC with TLM, such as complete exposure of the tumor, proper assessment tools with a suitable diagnostic workup, and having an experienced surgeon performing the procedure on this subsite of the larynx [69,70]. This is also suggested by the study of Vilaseca et al. [71], which investigated the impact on the AC involvement in patients with T1–T4a that were treated with TLM. They found that AC involvement was an independent factor for local recurrence. Half of their patients with recurrence were finally salvaged with TLM alone, suggesting that surgical experience could have played a role in local recurrence as a large proportion of patients were still amenable to TLM [71]. Tumors involving the AC and growing in the vertical plane are more difficult to expose due to a narrow angle, and the v-shaped configuration of the thyroid cartilage [54]. Difficult or incomplete surgical exposure has a tendency toward incomplete resection [61], which can subsequently lead to a higher recurrence rate. Several authors argue that tumors with vertical extension to the supra– and/or subglottic areas have a higher risk of local failure due to their narrow relationship with, and therefore the risk of (minor)spread into, the underlying visceral spaces [2,3].

To the best of our knowledge, no studies that treated patients with radiotherapy have used detailed stratification of the involvement of the AC. Therefore, although AC involvement, particularly in the vertical plane, may be a risk factor in TLM, it may well be the same for other treatment modalities. More studies are needed to investigate these factors in other treatment modalities to ascertain the relative benefits of different approaches.

### Limitations

The main limitation of this review is the heterogeneity of the studies that were included with regard to factors such as the clinical definition of the AC area, diagnostic protocols, and treatment protocols. Also, the majority of studies could not be included in the calculation of the weighted averages, as they did not present LC rates for patients with or without AC separately. Therefore, the weighted averages that were calculated should be interpreted with caution.

## 5. Conclusions

This review shows that the use of a binary variable (yes/no) for the involvement of the AC leads to conflicting results due to variability in definition, work-up, and treatment parameters of the AC area. However, weighted averages indicate that LC may be lower in tumors with involvement than tumors without involvement in the AC. Furthermore, all studies that use specific, detailed classifications of the AC show that there is a significant impact on outcome related to the amount of involvement of the AC. All in all, these findings point to a negative impact of AC involvement that may not be evident in simple binary (yes/no) studies of AC involvement. To further elucidate the role of the AC, detailed stratification of tumors involving the AC should be applied in future studies. To the best of our knowledge, no studies of patients treated with radiotherapy have used detailed stratification of the involvement of the AC. Therefore, to further elucidate the impact of AC involvement in these patients, stratifications need to be employed in these populations as well.

## Figures and Tables

**Table 1:** Oncological Outcomes of Patients after Treatment with Radiotherapy for Involvement with or without the AC.

First Author and Year	Treatment Period	Treatment Details	Tumor	Patients	Follow-up (in Months)	5-Year Local Control (%)	5-Year Overall Survival (%)	5-Year Disease Specific Survival (%)	5-Year Laryngeal Preservation (%)
Al-Mamgani 2014 [7]	1985–2011	Intended dose 66 Gy <’97 conventional >’98 accelerated	T1	AC+ 214 AC− 335	Median 93	OR = 1.1 *p* = 0.646			
Al–Mamgani 2013 [34]	1985–2011	Intended dose 66 Gy <’97 conventional >’98 accelerated	T1–T2	AC+ 553 AC− 497	Median 90	AC+ 84 AC− 86 OR 1.1 *p* = 0.091			
Berwouts 2016 [8]	2007–2011	IMRT: T1: 63 Gy T2: 67.5 Gy Conv RT T1: 66 Gy T2: 70 Gy	T1–T2	IMRT AC+ 7 AC− 33 Conv RT AC+ 11 AC− 70	IMRT: Median 45.6 Conv RT: Median 108	IMRT: *p* = 0.22 Conv RT: *p* = 0.62	IMRT: *p* = 0.60 Conv RT: *p* = 0.98	IMRT: *p* = 0.64 Conv RT: *p* = 0.27	
Bignardi 2004 [13]	1980–1988	63 Gy hyperfractionated	T2	AC+ 33 AC− 27	Median 117.6	HR 0.48 *p* = 0.12			
Bron 2001 [35]	1983–1996	Median 70 Gy	T1–T2	AC+ 43 AC− 38	Median 59	AC+ 66 AC− 90 *p* = 0.009		AC+ 88 AC− 100 *p* = NS	
Cellai 2005 [36]	1970–1999	<61 Gy 82 61–65 Gy 352 >65 Gy 397	T1	AC+ 282 AC− 549	Mean 111.6	AC+ 78 AC− 87 *p* = 0.001			
Cheah 2009 [37]	1993–2001	50 Gy	T1	AC+ 22 AC− 77	Median 84	AC+ 78 AC− 90 HR 2.17			
Chen 2003 [38]	1983–2001	T1: median 66 Gy T2: median 70 Gy	T1–T2	T1 AC+ 33 T1 AC− 55 T2 AC+ 29 T2 AC–17	Range 12–120	T1: 55 vs. 90 *p* = 0.0035 T2: 54 vs. 70 *p* = 0.74 MVA: RR 3.8 *p* = 0.020		T1 *p* = 0.0366	
Chera 2010 [14]	1964–2006	Median 63 Gy	T1–T2	AC+ 369 AC− 216	Median 147	*p* = 0.342			
Chung 2017 [15]	2006–2012	Median 65.25 Gy	Tis–T2	AC+ 52 AC− 112	Median 77.7	HR 1.67 *p* = 0.197			
Frata 2005 [39]	1970–1999	<61 Gy 33 61–65 Gy 83 >65 Gy 140	T2	AC+ 145 AC− 111	Mean 90	AC+ 69 AC–77 *p* = 0.1			
Garden 2003 [40]	1970–1998	Median 70 Gy	T2	AC+ 156 AC− 74	Median 82	AC+ 70 AC− 75 *p* = 0.59			
Gowda 2003 [41]	1989–1997	Total dose 50–52.5 Gy	T1	AC+ 50 AC− 150	Median 70	AC+ 89 AC− 94 *p* = 0.47			
Gultekin 2012 [42]	1998–2007	Median 64.4 Gy	T1	AC+ 31 AC− 152	Median 63	AC+ 79 AC− 82 *p* = 0.65	AC+ 78 AC− 92 *p* = 0.16	AC+ 81 AC− 92 *p* = 0.16	
Harada 2015 [16]	1999–2010	Hyperfractionated T1a: median 66 Gy T1b: median 70 Gy	T1–T2	AC+ 50 AC− 65	Median 61	UVA *p* = 0.25			
Jin 2002 [43]	1958–1994	Median 68.0 Gy	T1	AC+ 70 AC− 168	Median 127	AC+ 70.5 AC− 87.1 *p* = 0.003 HR 2.00 *p* = 0.024	AC+ 79.4 AC− 86.0 *p* = 0.32		
Jones 2010 [17]	1987–2006	T1 median 63 Gy T2 median 74.4 Gy	T1–T2	AC+ 70 AC− 48	Median 69.6	NS			
Khan 2012 [18]	1986–2006	Median 68.2 Gy	T1–T2	AC+ 71 AC− 52	Mean 67.2	UVA *p* = 0.0505 MVA *p* = 0.094			
Laskar 2012 [44]	1975–2000	Hypofractionated 50–62.5 Gy	T1	AC+ 228 AC− 414	Median 62	AC+ 86.3 AC− 90.3 *p* = 0.367			
Lim 2015 [45]	1981–2010	Median 66 Gy	T1–T2	AC+ 56 AC–166	Mean 85.2	AC+ 75.7 AC− 91.9 *p* < 0.001 MVA HR 3.37 *p* = 0.001			
Matsumoto 2016 [19]	2007–2014	Maximum total dose 63.0–70 Gy	T1–T2	AC+ 13 AC− 30	Median 33	UVA 0.085 MVA HR 4.97 *p* = 0.023			
Mendenhall 2001 [20]	1964–1998	Median 63 Gy	T1–T2	AC+ 328 AC− 191	Median 118.4	MVA *p* = 0.350	*p* = 0.224	*p* = 0.293	
Murakami 2005 [46]	1989–1998	T1a 60–66 Gy T1b–T2 64–70 Gy	T1–2	AC+ 59 AC− 71	Mean 75	AC+ 74 AC− 78 *p* = 0.668			
Nozaki 2000 [47]	1985–1997	Range 60–70 Gy	T1	AC+ 14 AC− 50	Not mentioned	AC+ 58 AC− 89 *p* < 0.05			
Raitola 2000 [9]	1970–1991	Range 45–70 Gy	T1–T2	AC+ 19 AC− 57	Median 82.8	HR 3.8 *p* = 0.004 MVA = NS		HR 3.0 (0.9–9.9) *p* = 0.0706	
Robert 2017 [48]	1987–2015	Mean 66.5 Gy	T1–T2	AC+ 45 AC–213	Median 50	AC+ 84 AC− 88 *p* = 0.382			
Sjogren 2009 [49]	1982–1993	Median 60 Gy	T1	AC+ 106 AC–210	Median 70	AC+ 85 AC− 87 *p* = 0.38			
Smee 2010 [10]	1967–2006	Median 60 Gy	Tis–T2	AC+ 127 AC− 395	Median 91.2	UVA *p* = 0.016 MVA *p* = 0.040		UVA = 0.019 MVA 0.050 (SE 0.303)	
Sommat 2017 [11]	2000–2012	Median 63.0 Gy	T1	AC+ 62 AC− 37	Median 58.8	HR 2.36 *p* = 0.274			
Thairat 2004 [12]	1975–2001	Median 66 Gy	Tis–T2	AC+ 37 AC− 118	Median 66	HR 1.1 *p* = 0.73			
Tong 2011 [50]	1983–2005	55–68 Gy	T1	AC+ 197 AC− 236	Median 126	AC+ 86 AC− 95 *p* = 0.004 MVA HR 2.34 *p* = 0.011			
Warde 1998 [51]	1981–1989	50 Gy	T1–T2	AC+ 261 AC− 474	Median 81.6	AC+ 75 AC− 85 *p* = 0.0005 MVA NS			
Yamazaki 2006 [5]	1993–2001	56.25–63 Gy	T1	AC+ 26 AC– 154	Median 64	OR 0.25 *p* = 0.25			
Zouhair 2004 [52]	1983–2000	Median 70 Gy	T1–T2	AC+ 61 AC− 61	Median 85	AC+ 73 AC− 94 *p* = 0.002 MVA RR 0.42 *p* = 0.001			

Abbreviations: AC+ = anterior commissure involvement, AC− = no anterior commissure involvement, AC0: no involvement of the anterior commissure, AC1 = involvement of the anterior commissure subsite on only one side of the midline, AC2 = involvement of the anterior commissure subsite that crosses the midline on only part of the longitudinal extension of this subsite, AC3 = involvement of the whole anterior commissure subsite on both sides across the midline, Conv RT = Conventional radiotherapy, Gy = Gray, HZ = hazard ratio, IMRT = Intensity Modulated Radiation Therapy, MVA = multivariate analysis, NS = not significant, OR = odds ratio, RR = Relative Risk, RT = radiotherapy, UVA = univariate analysis.

**Table 2:** Oncological Outcomes of Patients after Treatment with TLM for Involvement with or Without the AC.

First Author and Year	Treatment Period	Treatment Details	Tumor	Patients	Follow-up (in Months)	5-Year Local Control (%)	5-Year Overall Survival (%)	5-Year Disease Specific Survival (%)	5-Year Laryngeal Preservation (%)
Ansarin 2017 [53]	1999–2013	TLM (ELS I–VI)	Tis–T3	AC+ 102 AC− 483	Median 72	AC+ 79.4 AC –86.7 *p* = 0.04 MVA HR 1.29 *p* = 0.38		AC− 96.0 AC+ 87.2 *p* = 0.004	*p* = 0.12
Carta 2018 [54]	1993–2005 and 2010–2016	TLM (ELS I–VI)	Tis–T2	AC+ 105 AC–156 AC0 156 AC1 31 AC2 65 AC3 9	Median 51.6	AC+ 89.7 AC− 93.9 *p* = 0.205 AC0 93.9 AC1 96.2 AC2 89.3 AC3 74.1 *p* = 0.044	AC+ 78.6 AC− 89.5 NS	AC+ 98.4 AC− 100 NS	AC+ 95.3 AC− 99.1 *p* = 0.08 AC0 99.1 AC1 100 AC2 96.1 AC3 71.1 *p* < 0.0001
Chang 2017 [55]	2003–2009	TLM (ELS I–VI)	Tis–T3	AC+ 34 AC− 59	Median 35	AC+ 74 AC− 95 *p* = 0.007 MVA NS			
Chone 2007 [56]	1998–2003	TLM (ELS I–III)	T1–T2	AC+ 24 AC− 24	Mean 44	AC+ 79 AC− 96 *p* = 0.08			AC+ 96 AC− 100 *p* = 0.50
Fang 2013 [57]	2004–2011	TLM (ELS I–VI)	T1–T2	AC+ 45 AC− 28	Median 33	AC+ 83 AC− 85 *p* = 0.906			
Gallet 2017 [29]	2001–2010	TLM (ELS III–IV)	Tis–T2	AC+ 49 AC− 44	Median 75.6	UVA OR 3.4 *p* = 0.021 MVA = NS			
Hakeem 2013 [26]	2000–2011	TLM (nfs)	T1–T2	AC+ 61 AC− 235	Mean 49	*p* = 0.0001	AC+ 90.2 AC− 86.4 *p* = 0.642	AC+ 95.1 AC− 91.5 *p* = 0.642	AC+ 95.01 AC− 93.2 *p* = 0.287
Hartl 2007 [30]	1994–2006	TLM (ELS I–V)	Tis–T1	AC+ 8 AC− 79	Median 46	*p* = 0.16			
Hoffmann 2016a [58]	2001–2011	TLM (I–VI)	Tis–T2	AC+ 75 AC− 126	Mean 50.82	AC+ 54.6 AC− 79.8 *p* = 0.004	AC+ 76.9 AC− 88.5 *p* = 0.29	AC+ 90.8 AC− 99.0 *p* = 0.03	AC+ 91.9 AC− 100 *p* = 0.0003
Hoffmann 2016b [59]	2001–2013	TLM (Va–VI)	Tis–T2	AC1 29 AC2 17 AC3 50	Mean 44.3	AC1 71.6 AC2 87.5 AC3 50.8 *p*–0.04	NS	NS	NS
Hsin 2009 [27]	1999–2008	TLM (nfs)	Tis–T2	AC+ 18 AC− 30	Median 36.5	AC+ 74 AC− 71 *p* = 0.90			
Ledda 2006 [60]	1993–2001	TLM (ELS I–V)	Tis–T2	AC+ 22 AC− 81	Mean 70.8	AC+ 87.5 AC− 96.5 *p* = 0.6			
Lee 2013 [61]	1997–2011	TLM (ELS I–VI)	T1–T2	AC+ 33 AC− 85	Mean 69.4	AC+ 80.9 AC− 91.1 *p*= 0.583	AC+ 88.7 AC− 91.6 *p* = 0.883		
Mortuaire 2006 [33]	1990–2000	TLM (ELS I–V)	Tis–T2	AC+ 22 AC− 88	Median 42	UVA NS			
Peretti 2000 [62]	1987–1994	TLM (I–V)	Tis–T2	AC+ 40 AC− 98	Mean 76	AC + 72 AC− 86 *p* = 0.2			
Peretti 2001 [63]	1995–1997	TLM (I–V)	Tis–T1	AC+ 12 AC− 76	Mean 43	AC+ 83 AC− 87 *p* = 0.7			
Peretti 2010 [3]	1988–2005	TLM (ELS I–V)	Tis–T1	AC+ 84 AC− 391	Mean 84	AC+ 100 AC–99.2 *p* = 0.44		AC+ 100 AC− 98.9 *p* = 0.27	AC+ 98.8 AC− 98.1 *p* = 0.57
Peretti 2013 [64]	2005–2010	TLM (ELS Type V)	T2–T3	AC+ 4 AC− 85	Minimal 18	AC+ 59 AC− 62 NS			AC+ 96 AC− 75 NS
Rodel 2009 [28]	1986–2004	TLM (nfs)	T1–T2	T1a AC+ 55 T1a AC− 237 T1b AC+ 34 T1b AC− 16 T2 AC+ 64 T2 AC− 38	Median 65	T1a: 73 vs. 89 *p* = 0.06 T1b: 68 vs. 86 *p* = 0.32 T2: 76 vs. 76	T1a: 85 vs. 87 T1b: 93 vs. 72 T2: 80 vs. 59 NS		T1a: 95 vs. 98 T1b: 88 vs. 100 T2: 89 vs. 95 no *p*–value
Rucci 2010 [31]	2003–2007	TLM (ELS I–V)	Tis–T1	AC0 48 AC1 20 AC2 13 AC3 0	Mean 24.2	UVA *p* =0.0119 MVA OR 5.14 *p* = 0.036			
Sachse 2009 [65]	1995–2005	TLM (ELS II–Va)	T1	AC+ 14 AC− 32	Median 36	AC+ 42 AC− 87 NS	AC+ 67 AC− 100		
Son 2018 [32]	2009–2014	TLM (ELS I–VI)	T1–T2	AC+ 25 AC− 48	Median 44	HR 3.45 *p* =0.030 MVA 1.03 *p* = 0.964	1.96 *p* = 0.412		
Steiner 2004 [23]	1986–1996	TLM (proposal by Steiner)	T1–T2	T1a AC+ 28 T1a AC− 130 T1b AC+ 16 T1b AC− 14 T2 AC+ 45 T2 AC− 30	Median 63.9	T1a: 84 vs. 90 T1b: 73 vs. 92 T2: 79 vs. 74 All *p*–value > 0.05	T1a: 87 vs. 86 T1b: 100 vs. 70 T2: 80 vs. 56 no *p*–value		*T1a: 93 vs. 99* *T1b: 88 vs. 100* *T2: 93 vs. 97* no *p*–value
Wolber 2017 [24]	1992–2002	TLM (proposal by Steiner)	T1–T2	AC+ 21 AC–28	Mean 62.0	AC+ 57.1 AC− 92.9 *p* < 0.01	AC+ 90.5 AC− 96.4 *p* = 0.39		

Abbreviations: AC+ = anterior commissure involvement, AC− = no anterior commissure involvement, AC0 = no involvement of the anterior commissure, AC1 = involvement of the anterior commissure subsite on only one side of the midline, AC2 = involvement of the anterior commissure subsite that crosses the midline on only part of the longitudinal extension of this subsite, AC3 = involvement of the whole anterior commissure subsite on both sides across the midline, ELS = European Laryngology Society, HZ = hazard ratio, MVA = multivariate analysis, nfs = not further specified, NS = not significant, OR = odds ratio, TLM = transoral laser microsurgery, UVA = univariate analysis.

**Table 3:** Weighted Averages for 5-Year Local Control Classified by Tumor Group.

Heading Title		*n*	T1	*n*	T2	*n*	T1–T2
**Radiotherapy**	AC+ AC–	1033 2064	82.2 89.1	330 202	68.2 75.7	1140 1592	78.2 86.8
**TLM**	AC+ AC–	147 429	70.1 89.1	109 68	77.2 75.1	123 165	77.3 91.1
